# The relationship between body mass index (BMI) and quality of life in Iranian primary school students in Tehran, Iran

**DOI:** 10.1051/bmdcn/2018080103

**Published:** 2018-02-26

**Authors:** Mostafa Farajpour. kh, Mohadeseh PishgahRoodsari, Hamid Salehiniya, Fahimeh Soheilipour

**Affiliations:** 1 Minimally Invasive Surgery Research Center, Iran University of Medical Sciences Tehran Iran; 2 Zabol Medical Science University Zabol Iran; 3 Epidemiology and Biostatistics Department, School of Public Health, Tehran University of Medical Sciences Tehran Iran; 4 Pediatric growth and development research center ,institute of endocrinology and metabolism, Iran University of Medical Science Tehran Iran

**Keywords:** Body mass index, Children, Obesity, Quality of Life, Iran, Students

## Abstract

Background: This study aimed to investigate the relationship between Body Mass Index (BMI) and quality of life in primary school students in Tehran.

Method: In this cross-sectional study 829 primary school children and their parents participated. Healthrelated quality of life (HROOL) was evaluated with the Persian version of Pediatric Quality of Life Inventory (PedsQL^™^4.0) questionnaire. According to objective measures of height and weight, children BMI computed, and adapted for age and gender. For data analysis we used Pearson correlation test, Independentsample *t*-test and ANOVA using SPSS version 18.

Results: Mean of children self-reported HRQOL total score was 82.05 ± 12.04 and mean of parent proxyreported HRQOL total score was 81.66 ± 12.81. Based on HRQOL subscale scores, social functioning was the highest subscale score of HRQOL (84.67 ± 15.07) and the emotional subscale score was the lowest (77.79 ± 17.26). Lower HRQOL scores were significantly correlated with Higher BMI and normal weight children had significantly higher HRQOL total score than obese children (*P* < 0.05). The difference between normal weight and overweight children in HRQOL total scores were not significant. Same results were obtained from parent proxy-reports and a good harmony between children self-report and parent proxy-report of HRQOL was perceived.

Conclusion: This study showed that HRQOL of obese children were at the lower level in comparison to normal weight and overweight children. At further interventional studies these outcomes can be very important for improving quality of life in obese children.

## 1. Introduction

Nowadays, one of the most important world health concerns is the increased rate of obesity and overweight through childhood [1, 2] Based on CDC 2000 BMI standards; In 2012 among ages 2-19, 31.8% of them were overweight and 16.9% were obese.[3]

Based on WHO-HBSC examine, overweight prevalence is more than 10% among school-developed children in most countries, with an extent of 7.6% (Latvia) to 28.8% (USA) [4]. Obesity and overweight of children are serious concerns in Iran and prevalence of obesity in children are at high incidence in this country.[5, 6]

Childhood overweight and obesity are related to advancement of several long-term diseases such as CAD (coronary artery disease), DM (diabetes mellitus), metabolic syndrome, stroke, dyslipidemia, hypertension and asthma [7-10]. Likewise, childhood obesity is known as a risk factor of adulthood obesity [11]. Obesity also causes many mental disorders, for example, low confidence, gloom, body disappointment, impaired social relationships and obesity stigma in young people [12-15].

One of the most important issues that are raised in relation to obesity and overweight, is quality of life. Quality of life can be characterized as a multidimensional develop that mirrors one's self-view of pleasure and contentment [16]. Several studies indicate the relation between BMI and HRQOL, so that in obese and overweight persons with quality of life at a lower level and is associated with stress and mental disorders [17-19].

Among elementary school understudies in Iran, there was a study with the aim of exploring the relationship between body mass Index (BMI) and Quality of life (Khodaverdi, Alhani, Kazemnejad, & Khodaverdi, 2011). The result confirmed that HRQOL particularly in physical, social and school functioning subscales in obese children were at the lower level in comparison with their ordinary weight matches.

Some studies have shown the strong relationship between obesity and HRQOL and some others not, there is not definitive and comprehensive study in this field in Iran. By the importance of the subject, the aim of this study was to examine the correlation between body mass index (BMI) and HRQOL in primary school students in Tehran in 2016.

## 2. Materials and methods

### 2.1. Study design and sampling

This cross-sectional study was conducted among elementary school students with the age of aged 8 to12 year old in Tehran in 2016. The multi-stage sampling was used to select subjects. Firstly, Tehran was divided into five areas of the South, East, North, West and Central division. Then the Department of Education of each zone was visited and a list of non-profit and for-profit schools was observed, two elementary chosen selected using simple random sampling method from each zone, thus our study was conducted in a total of ten elementary schools.

The duration of primary schools in Iran is six years, we selected six classes from each school in order to participate in this study. Of course students with any chronic disease like Malignancies, Diabetes and kidney disease were excluded from study.

### 2.2. Measurements

#### 2.2.1. Assessment of weight status

Students' height and weight were measured in collaboration with the school health educators and trained nurses. Students had minimum clothes and no shoes during scaling with the normal position of the shoulder. BMI was computed by the ratio of weight (Kg) to height squared (m^2^).

MedCalc 3000 system was used to decide body weight status of students. According to student's age, BMI was categorized into four groups, including underweight (<5 percentile), healthy weight (≥5 percentile to <85 percentile), overweight (≥85 percentile to <95), obesity (≥95 percentile). [21]

### 2.3. Health-related quality of life (HRQOL)

For evaluation HRQOL of children, the Persian version PedsQL ™ 4.0 questionnaire was used. The reliability and validity of the scale have been Repeatedly confirmed (α = 0.71-0.89). [22]

The PedsQL™ 4.0 is a self-administered questionnaire consists of Generic Core Scales that are comprised of parallel child selfreport and parent proxy-report formats and includes 23 items.

It envelops four subscales physical functioning, emotional functioning, social functioning, and school functioning. Physical subscale consists of five categories: limitations with effortful movements (like sports), (b) constraint of regular exercises, (c) physical symptoms like pain, (d) feelings about physical wellbeing and physical sensations, and (e) acute and chronic disorders like Asthma, epilepsy, diabetes, *etc*.

Emotional subscale consists of five categories: (a) negative feelings (feeling stressed, feeling sad and having sleep issues), (b) positive emotions (Feeling happy and cheerful), (c) confidence and body image, (d) cognitive functioning (Difficulty focusing, recollect those learned at school), and (e) general behavior.

Social functioning subscale refers to peer relationship, problems with companion and capacity to make companions.

School functioning subscale refers to cognitive functioning such as trouble in focusing and association with teachers. [23]

A five-point scale (from 0 = never a problem to 4 = almost always a problem) was used for both children self-report and parent proxy-report, items were scored reversely (0 = 100, 1 = 75, 2 = 50, 3 = 25 and 4 = 0); so, a higher total score shows a better HRQOL. [24]

### 2.4. Data collection

First, the study protocol was approved by the Medical Research Ethical Committee of Obesity Research Center of Minimally Invasive Surgery, Iran University of Medical Science.

Then, the approvals were obtained from the Ministry of Education in Tehran and selected schools. Oral assents and signed consents were obtained from both children and their parents, and questionnaires were distributed to them. All questionnaires were completed anonymously.

Totally, 920 children and their parents were participated in this study. 53 students were excluded from study because of chronic diseases (2 for anemia,1 for migraine, 1 for CKD,1 for kidney stone,5 for heart disease, 2 for G6PD, 2 for recurrent UTI, 1 for nocturia, 2 for chronic sinusitis, 2 for GER, 3 for diabetes, 2 for hypothyroidism, 2 for epilepsy, 1 for thalassemia, 11 for asthma and 15 for allergies), 13 students were excluded because of incomplete information. Therefore, a total of 829 children and their parents participated in this study. After children’s selfreport version of HRQOL questionnaires were distributed to the children, they were made a request to bring back finished parentproxy sheet of HRQOL questionnaire on the following day. A Rule was set up to clarify the method for filling in parent-proxy version of HRQOL questionaires.

### 2.5. Data analysis

We used SPSS (Statistical Package for Social Sciences, V 11.5) program for Statistical analysis. Mean and standard deviation were calculated for quantitative variables and frequency (percent) describes qualitative variables. Pearson correlation analysis was used to examine the correlations between BMI or age and scores of PedsQL. Independent-samples *t*-test was used to compared total and subscale scores of PedsQL between two sexes. One-way ANOVA test was used to determine differences between groups of body weight status. Fisher’s Least Significant Difference (LSD) test as post hoc test was used to determine which four groups of body weight status were significantly difference in PedsQL scores. Less than 0.05 was accepted as indicating statistical significance.

### 2.6. Result

Totally 829 students were enrolled in this study, with mean age of 9.76 ± 1.48 (Rang: 8-12 years). Among included children, 463 (55.9%) were girls. The mean BMI of children were 18.60±4.02 (18.12 ± 3.72 and 19.20 ± 4.32 in girls and boys respectively; *P* < 0.001). The body weight status of children were classified to 61 (7.4%) as underweight, 476 (57.4%) as normal weight, 171 (20.6%), as overweight and 121 (14.6%) as obese. Table 1 shows demographic data of the children.

**Table 1:** General characteristics of the 829 children among school students of Tehran.

Variables	Total	Girl	Boy
Age, mean (SD), yr	9.76 ± 1.48	9.57 ± 1.41	10.02 ± 1.53
Height ,mean (SD), cm	138.69 ± 11.85	136.97 ± 11.04	140.87 ± 12.47
Weight ,mean (SD), kg	36.43 ± 11.55	34.56 ± 10.48	38.79 ± 12.39
BMI ,mean (SD)	18.60 ± 4.02	18.12 ± 3.72	19.20 ± 4.32
BMI group			
Underweight	61 (7.4)	35 (7.6)	26 (7.1)
Normal weight	476 (57.4)	282 (60.9)	194 (53)
Overweight	171 (20.6)	96 (20.7)	75 (20.5)
Obese	121 (14.6)	50 (10.8)	71 (19.4)

Table 2 shows mean of children self-reported and parent proxy-reported total and subscale scores of PedsQL. There were no significant differences between mean of children self-reported and parent proxy-reported total and subscale scores (for all of them, *P* > 0.05). Based on both children self-reported and parent proxy-reported, social functioning was the highest subscale score (84.67 ± 15.07 and 85.13 ± 15.29, respectively) and emotional functioning was the lowest subscale score (77.79 ± 17.26 and 76.45 ± 17.68, respectively).

**Table 2:** Comparison between Children self-reported and Parent proxy-report of PedsQL§.

	children self-report	Parent proxy-report	P-value
Physical functioning subscale score	82.84 ± 14.60	83.17 ± 14.90	0.648
Emotional functioning subscale score	77.79 ± 17.26	76.45 ± 17.68	0.120
Social functioning subscale score	84.67 ± 15.07	85.13 ± 15.29	0.535
School functioning subscale score	82.87 ± 15.83	81.86 ± 16.99	0.212
Total Score	82.05 ± 12.04	81.66 ± 12.81	0.519

§ Independent-samples *t*-test was used.

According to gender classification, based on children selfreported scores of PedsQL, school functioning was significantly higher in girls compared with boys (84.52 ± 14.49 vs. 80.78 ± 17.17, *P* = 0.001) while other subscales and total score were the same in girls and boys children (for all of them, *P* > 0.05) (Table 3).

**Table 3:** Comparison of children self-reported subscale scores and total score of PedsQL based on sex§.

	Girls	Boys	P-value
Physical functioning subscale score	82.66 ± 14.39	83.05 ± 14.88	0.706
Emotional functioning subscale score	77.04 ± 17.49	78.64 ± 16.94	0.160
Social functioning subscale score	85.45 ± 14.93	83.68 ± 15.20	0.093
School functioning subscale score	84.52 ± 14.49	80.78 ± 17.17	0.001
Total Score	82.42 ± 11.74	81.58 ± 12.40	0.314

§ Independent-samples *t*-test was used.

Data analysis revealed no significant correlation between children’s age and self-reported total score of PedsQL (r = 0.05, *P* = 0.156) and none of all self-reported subscale scores such as physical (r = 0.06, *P* = 0.093), emotional (r = -0.01, *P* = 0.668), social (r = 0.07, *P* = 0.059) and school (r = 0.05, *P* = 0.152); also, the correlation between children’s age and parent proxy-reported total score (r = 0.05, *P* = 0.113) and all subscale scores except social score (r = 0.7, *P* = 0.033), such as physical (r = 0.03, *P* = 0.319), emotional (r = -0.001, *P* = 0.987), and school (r = 0.07, *P* = 0.60) functioning score were not significant (data is not showing in table).

As shown in table 4, the association between BMI and selfreported total score of PedsQL (r = -0.109, *P* = 0.038), physical (r = -0.134, *P* = 0.01), and social (r = -0.115, *P* = 0.028) were negatively significant among boys, whereas no statistically significant correlation were observed in the emotional (r = -0.084, *P* = 0.110) and school (r = -0.017, *P* = 0.747) subscale score.

**Table 4:** Correlation coefficients (r) for BMI and children self-reported total and subscale scores of PedsQL§.

	BMI		
	
	Boys	Girls	Total
Physical functioning subscale score	-0.134*	-0.012	-0.069*
Emotional functioning subscale score	-0.084	+0.007	-0.029
Social functioning subscale score	-0.115*	-0.047	-0.087*
School functioning subscale score	-0.017	-0.067	-0.056
Total Score	-0.109*	-0.037	-0.076*

§ Pearson Correlation coefficients was used.

* *P*-value < 0.05.

In girls, there is no significant correlation between BMI and self-reported total score of PedsQL (r = -0.037, *P* = 0.433) and self-reported subscales scores such as physical (r = -0.012, *P* = 0.796), emotional (r = 0.007, *P* = 0.889), social (r = -0.047, *P* = 0.311) and school (r = -0.067, *P* = 0.151) (Table 4).

In overall, the correlation coefficient between BMI and total score of children self-reported (r = -0.076, *P* = 0.029), physical (r = -0.069, *P* = 0.047), and social functioning score (r = -0.087, *P* = 0.013) were significantly negative. Although the correlation between BMI and emotional (r = -0.029, *P* = 0.403) and school functioning score (r = -0.056, *P* = 0.109) are negative, they were not statistically significant (Table 4).

Table 5 shows self-reported total and subscale scores of PedsQL according to BMI classification, normal weight children had highest mean total score of PedsQL and all subscale scores compared with underweight, over weight and obese groups; whereas the mean score for total, physical and social subscale scores were significantly different by body weight status (*P* < 0.05, for all of them) (table 5). The result of post hoc test for total score of PedsQL showed that the difference between normal weight and obese were statistically significant (*P* = 0.004). In physical subscale score, obese (*P* = 0.004) and overweight children (*P* = 0.006) had lower mean scores compared with normal weight children; furthermore, social subscale score in obese children had significantly lower mean score than normal weighted group (*P* = 0.003).

**Table 5:** Mean ± SD PedsQL by BMI category§.

Total	BMI category				

	Underweight	Normal	Overweight	Obese	P-value
Physical functioning subscale score	81.57 ± 15.42	84.41 ± 13.15	80.87 ± 16.27^a^	80.09 ± 16.40^a^	0.04
Emotional functioning subscale score	75.96 ± 19.22	78.60 ± 16.27	77.57 ± 18.36	75.83 ± 18.39	0.345
Social functioning subscale score	83.83 ± 15.71	85.80 ± 13.62	84.27 ± 16.81	81.20 ± 17.06^a^	0.025
School functioning subscale score	80.82 ± 17.14	83.82 ± 14.82	82.20 ± 16.44	81.12 ± 17.90	0.214
Total Score	80.55 ± 13.09	83.15 ± 10.56	81.28 ± 13.73	79.58 ± 13.87^a^	0.013
**Girl**					
Physical functioning subscale score	78.05 ± 17.64^a^	84.30 ± 12.70	79.74 ± 15.92^a^	82.32 ± 16.47	0.01
Emotional functioning subscale score	73.11 ± 18.36	77.87 ± 16.45	74.92 ± 19.06	79.10 ± 19.16	0.213
Social functioning subscale score	83.57 ± 16.43	86.28 ± 13.58	84.18 ± 16.77	84.57 ± 17.34	0.517
School functioning subscale score	82.50 ± 15.25	85.51 ± 13.40	81.83 ± 16.01	85.51 ± 16.40	0.137
Total Score	79.31 ± 13.91^a^	83.49 ± 10.10	80.18 ± 13.87^a^	82.92 ± 13.56	0.038
**Boy**					
Physical functioning subscale score	86.31 ± 10.33	84.56 ± 13.81	82.31 ± 16.69	78.51 ± 16.28^a^ ^c^	0.018
Emotional functioning subscale score	79.81 ± 20.02	79.65 ± 15.97	80.93 ± 16.98	73.52 ± 17.59^a^ ^b^	0.033
Social functioning subscale score	84.18 ± 14.99	85.10 ± 13.68	84.39 ± 16.98	78.78 ± 16.56^a^ ^b^	0.027
School functioning subscale score	78.56 ± 19.47	81.34 ± 16.40	82.69 ± 17.09	78.10 ± 18.37	0.352
Total Score	82.21 ± 11.97	82.65 ± 11.20	82.69 ± 13.52	77.23 ± 13.69^a^ ^b^	0.012

§ One-Way ANOVA was used; for *post hoc* test Fisher's Least Significant Difference (LSD).

a : compared with normal weight group, *p* < 0.05.

b : compared with overweight group, *p* < 0.05.

c : compared with underweight group, *p* < 0.05.

In girls, self-reported total and physical scores of PedsQL were significantly different by body weight status (for both of them, *P* < 0.05). The result of post hoc test for total score in underweight (*P* = 0.046) and overweight (*P* = 0.017) girls was significantly lower than normal weighted, the same result was found in the physical functioning subscale, so underweight (*P* = 0.015) and overweight (*P* = 0.007) girls had significantly lower mean score compared to normal weighted.

In boys, the mean score for total and all subscale scores of PedsQL were significantly different by BMI category (*P* < 0.05, for all of them), except for school functioning subscale (*P* = 0.352) (table 5). Comparing obese boys with normal weight and overweight groups, there is significantly lower total score (*P* = 0.002 and *P* = 0.007, respectively), emotional (*P* = 0.003 and *P* = 0.026, respectively) and social score (*P* = 0.009 and *P* = 0.008, respectively); in physical functioning subscale score, obese boys had significantly lower mean score compared to normal weight (*P* = 0.003) and underweight (*P* = 0.022) ones.

Based on parent proxy reports in overall, as body weight status, total score of PedsQL, and all subscale scores (*P* < 0.05, for all of them) except physical subscale score (*P* = 0.242) were significantly different (Table 6). Result of post-hoc tests, obese children compared to normal weight and overweight groups had lower PedsQL in total score (*P* < 0.001 and *P* = 0.035, respectively), emotional score (*P* = 0. 01 and *P* = 0.028, respectively), and social subscale scores (*P* < 0.001 and *P* = 0.031, respectively). Additionally, in school functioning subscale the difference between normal weight and obese children is statistically significant (*P* = 0.008).

**Table 6:** Mean ± SD of parent-proxy reporting on the PedsQL by BMI category§.

Total	BMI category				

	Underweight	Normal	Over weight	Obese	P-Value
Physical functioning subscale score	82.63 ± 16.47	84.01 ± 13.75	82.10 ± 15.66	81.25 ± 17.13	0.242
Emotional functioning subscale score	74.20 ± 21.51	77.42 ± 16.50	77.48 ± 18.16	72.77 ± 19.03^a^ ^b^	0.045
Social functioning subscale score	83.28 ± 17.79	86.86 ± 13.17	84.22 ± 17.11	80.76 ± 17.57^a^ ^b^	0
School functioning subscale score	79.45 ± 17.45	83.23 ± 15.69	81.28 ± 18.74	78.81 ± 17.74^a^	0.031
Total Score	79.89 ± 14.70	82.83 ± 11.06	81.44 ± 14.68	78.25 ± 14.71^a^ ^b^	0.003
**Girl**					
Physical functioning subscale score	81.25 ± 17.08	83.81 ± 13.42	82.15 ± 14.96	83.03 ± 16.77	0.664
Emotional functioning subscale score	72.46 ± 20.35	76.94 ± 16.89	75.42 ± 18.09	76.25 ± 19.14	0.503
Social functioning subscale score	83 ± 18.32	87.98 ± 12.59	84.09 ± 16.70	85.65 ± 16.67	0.07
School functioning subscale score	80.96 ± 16.73	84.70 ± 14.59	80.57 ± 17.85	83.50 ± 15.56	0.118
Total Score	79.42 ± 14.71	83.36 ± 10.57	80.56 ± 14.33	82.11 ± 13.74	0.118
**Boy**					
Physical functioning subscale score	84.49 ± 15.75	84.29 ± 14.25	82.03 ± 16.62	79.97 ± 17.38	0.231
Emotional functioning subscale score	76.54 ± 23.18	77.98 ± 15.96	80.14 ± 18.08	70.28 ± 18.55^a^ ^b^	0.004
Social functioning subscale score	83.65 ± 17.41	85.23 ± 13.86	84.38 ± 17.73	77.27 ± 17.48^a^ ^b^	0.003
School functioning subscale score	77.40 ± 17.50	81.08 ± 16.97	82.19 ± 19.91	75.46 ± 20.16	0.07
Total Score	80.52 ± 14.94	82.06 ± 11.71	82.44 ± 15.15	75.53 ± 14.86^a^ ^b^	0.03

§ One-Way ANOVA was used; for *post hoc* test, Fisher's Least Significant Difference (LSD).

a : compared with normal weight group, *p* < 0.05.

b : compared with overweight group, *p* < 0.05.

In girls, according to parent proxy-reported total score and all subscale scores of PedsQL were not significantly different by BMI category (for all of them, *P* > 0.05). This finding shows that parents perceived PedsQL of girls were similar regardless of their BMI.

Based on parental perception of PedsQL in boys, obese children compared to normal weight and overweight children, had the lower scores in total score (*P* < 0.001 and *P* = 0.002, respectively) emotional (*P* = 0.002 and *P* = 0.001, respectively) and social (*P* < 0.001 and *P* = 0.004, respectively).

## 3. Discussion

In this study, we evaluate the relationship between HRQOL and BMI of primary school children. Our findings showed that obese children had lower HRQOL than other children.

The mean total score HRQOL self-reported among children were 82.05 ± 12.04 and parent proxy-reported were 81.66 ± 12.81 in this study. Self-reported scores are similar to parent-proxy scores and are higher than scores that observed by Amiri *et al.* among school children of Tehran in 2012 (self-reported score: 83.99 ± 11.90, parent proxy-reported score: 76.75 ± 13.45). [25] There was a study by Hovsepian *et al.* in 2011 during a nationwide study in Iran (the Weight Disorders Survey of the CASPIAN-IV study) investigated 23,043 students (6-18 years) and mean total PedsQL™ score in children (age 6-12 years ) was 83,79. [26]

In another study conducted in Qazvin (a city near to Tehran) in 2011, among 1107 students aged between 8and 18, children self-reported was 77.55 ± 13.02 and parent proxy report was 75.77 ± 18.42. [27]

It appears that total mean PedsQL™ score is comparative in different areas of Iran using the same score scale. The finding by Hovsepian *et al.* (2017) [26]

In this study there was a harmony between children selfreport and parent proxy-report of HRQOL which is in agreement with the results of study by Amiri *et al.* (2012) high parental support in Iranian families can explain the harmony between children self-report and parent proxy-report of HRQOL. [25] this finding is not consistent with results of studies in most of the other translated versions. [28-30]

Based on our results, emotional and social functioning subscales had the lowest and the highest HRQOL subscale scores respectively. In Previous studies, Iranian children had the lowest HRQOL score in the subscale of emotional functioning while the highest HRQOL subscale score of children was in social functioning [25]. A good capacity in making companions and peer relationships of Iranian children can explain these findings. On the other hand, emotional functioning was the poorest subscale in comparison with the three other subscales of HRQOL. This finding indicates that negative emotions and rest related issues are the most well-known issues among Iranian children and these health problems should be considered by health-care professionals. [31]

In our study, age was not related to HRQOL in primary school children of Tehran. Although in previous studies (in children 8-18) HRQOL declined with age [32] this finding could be due to selecting participant from a narrow range of age in this study. (children 8-12)

Past examines have exhibited sexual orientation contrasts in the pediatric HRQOL. [22, 27]

Our study showed that girls had significantly higher HRQOL in school functioning score than boys, by self-report. However, boys in emotional and social functioning had higher scores, but the differences weren’t significant. Wynne *et al.* in 2014 showed same results in Ireland. [33] Amiri *et al.* [25] showed that girls have significantly higher scores in all subscales. Pakpour *et al.* in another study in 2011 demonstrated that boys have higher scores but differences weren’t significant [27]. In previous studies, among adolescents, girls have lower scores in all subscales. The difference between adolescents and children might be due to the diverse impression of social and ecological weight and furthermore to pubescence changes in teenagers. [25]

In general, it can be concluded that by increasing age (From childhood to adolescence) the quality of life (especially in social domain) in obese children due to social problems decreases. [26]

BMI for age was inversely correlated to HRQOL total score in this study. Also HRQOL scores in normal weight children were significantly higher than obese children. Our results consisted of past investigations which demonstrate opposite impact of obesity on HRQOL. [34]

The decline in HRQOL score was not significant for overweight children in comparison with normal weight children, but it was significant for those who were obese, that is in agreement with the results reported by Khodaverdi *et al.* in 2011 [20] poorer HRQOL scores in obese children in comparison with normal weights children can be a cause of negative effects of obesity outcomes. Also, higher BMI can predict higher levels of disordered eating attitudes and behaviors, which lead to lower levels of HRQOL. [35]

Since the HRQOL is multidimensional, it is sensible that some domains might be more influenced by overweight or obesity. Various results have been obtained in various studies in this field. For example, Williams *et al.* demonstrated that aggregated score, physical health, and social functioning score diminished significantly as weight increased [36]. In another study overweight children had significantly lower scores on psychosocial, physical functioning and total scores in comparison with normal weight children. [37]

In a study by Fiveash *et al.*, obese children had Lower scores in social functioning compared to underweight, normal weight and overweight [38] another examination demonstrated that obese children were over five times more likely to report poor quality of life scores when in comparison with normal weight children. [17]

In another study by Pinhas-Hamiel *et al.*, demonstrated that obese children significantly poorer quality of life related to physical functioning and social domains compared to normal weight children. [18] In an investigation of clinical sample of obese children, they had significantly poorer physical health compared to control group. [39]

In our study, by physical dimension, obese and overweight children had significantly lower scores than normal weight children. This finding maybe be related to restrictions associated with excess weight for obese and overweight children (like difficultly running and doing exercise), however, they were not so heavy as to seek medical treatment [20]. It could be in mind that in another study physical dimension were not different between weight groups. [38]

BMI was conversely associated with physical functioning in this study. This supports the idea that reduced activity can make mismatch between production and consumption calories and will lead to weight gain. [17]

In social dimension, obese children had significantly lower scores. This finding elucidates that Obesity is one of the most disparaging and least socially adequate conditions in childhood [17] Obese children are exposed to labeling of obese and ridicule and bullying from other students. [40]

On the other hand, obese children had significantly lower scores in the social domain, but the difference between overweight and normal weight children isn’t significant. So slight decrease in weight (i.e., shifting from obese to overweight) may improve social wellbeing. This finding is consistent with khodaveri *et al.* [20]

In Hovsepian S *et al.* study, in physical and social domain, there was no significant difference between weight groups. [26]

In emotional and school functioning, these relations weren’t significant and emotional and school functioning dimension seem unaffected.

Although in Hovsepian S *et al.* study, emotional and school functioning domains had a negative association with obesity. [26]

The lack of differences in emotional subscale scores across weight categories is consistent with khodaverdi *et al.* [20] this can revealed that obese children despite physical and social difficulties, keep up their emotional health and endeavors for understanding the nature and determinants of constancy to create preventive interventions to advance HRQOL of them will be useful. [41]

It appears that different sample size of studies and also cultures of different societies may cause this variety of outcomes in several studies. In addition, in this sample of study, overweight children is higher than obese ones, and given that overweight children have less difficulty in quality of life (especially in the physical domain) compare to obese children, the effect of weight gain on quality of life may not well defined. [26]

Using a valid instrument for evaluation HRQOL was the main quality of this study. On the other hand, this is a generic instrument. The generic instrument may be less sensitive than specific instruments for obesity to evaluate the relationship between body weight status and HRQOL of the children. However, PedsQL is very popular for assessing HRQOL in many countries. It examines the quality of life from the perspective of children and their parents, and allows us to compare them.

There are also some limitations in this study. We can't make a causal assessment about connections amongst obesity and HRQOL due to its cross-sectional nature.

In conclusion, our results demonstrated that obesity was associated with poor HRQOL in children of primary schools of Tehran. Future investigations should be concentrated on obesityrelated practices that may add to poor HRQOL among Iranian children, in addition, obese children have lower HRQOL scores especially physical and social dimensions. Further studies should consider the importance such dimensions in future. On the other hand, the comprehension of the flexibility of obese children in emotional and school functioning dimension may help in approaches to manage the administration of childhood obesity.

## Conclusion

Our results demonstrated that obesity was associated with poor HRQOL in children of primary schools of Tehran.

Obese children have lower HRQOL scores especially in physical and social domains but emotional and school functioning domains seemed unaffected.

## Declaration of Conflicting Interests

The author(s) declared no potential conflicts of interest with respect to the research, authorship, and/or publication of this article.

## References

[1] Black MM, Hager ER, Le K, Anliker J, Arteaga SS, DiClemente C, et al. Challenge! Health promotion/obesity prevention mentorship model among urban, black adolescents. Pediatrics. 2010: peds. 2009-1832. 10.1542/peds.2009-1832 PMC4124131 20660556

[2] Chandra AR. An Investigation of Advertising on Media, Socio Economic, Gender, And Age Relationship With Obesity. POLI BISNIS. 2012; 3: 1-11.

[3] Ogden CL, Carroll MD, Kit BK, Flegal KM. Prevalence of childhood and adult obesity in the United States, 2011-2012. Jama. 2014; 311: 806-14. 24570244 10.1001/jama.2014.732 PMC4770258

[4] Haug E, Rasmussen M, Samdal O, Iannotti R, Kelly C, Borraccino A, et al. Overweight in school-aged children and its relationship with demographic and lifestyle factors: results from the WHO-Collaborative Health Behaviour in School-aged Children (HBSC) study. Int J Public Health. 2009; 54:167-79. 19618111 10.1007/s00038-009-5408-6 PMC2735089

[5] Kelishadi R, Hashemi Pour M, Sarraf-Zadegan N, Ansari R, Alikhassy H, Bashardoust N. Obesity and associated modifiable environmental factors in Iranian adolescents: Isfahan Healthy Heart Program-heart health promotion from childhood. Pediatr Int. 2003; 45: 435-42. 12911481 10.1046/j.1442-200x.2003.01738.x

[6] Dorosty AR, Siassi F, Reilly JJ. Obesity in Iranian children. Arch Dis Child. 2002;87(5):388-91; discussion-91. 12390907 10.1136/adc.87.5.388 PMC1763078

[7] Weiss R, Dziura J, Burgert TS, Tamborlane WV, Taksali SE, Yeckel CW, et al. Obesity and the metabolic syndrome in children and adolescents. N Engl J Med. 2004; 350: 2362-74. 15175438 10.1056/NEJMoa031049

[8] Ho TF. Cardiovascular risks associated with obesity in children and adolescents. Ann Acad Med Singapore. 2009; 38: 48. 19221671

[9] Ford ES. The epidemiology of obesity and asthma. J Allergy Clin Immunol. 2005; 115: 897-909. 15867841 10.1016/j.jaci.2004.11.050

[10] Daniels SR. The consequences of childhood overweight and obesity. Future Child. 2006:47-67. 16532658 10.1353/foc.2006.0004

[11] Rossner S. Childhood obesity and adulthood consequences. Acta paediatrica. 1998; 87: 1-5. 9510438 10.1080/08035259850157778

[12] Strauss RS, Pollack HA. Social marginalization of overweight children. Arch Pediatr Adolesc Med. 2003; 157: 746-52. 12912779 10.1001/archpedi.157.8.746

[13] Wang F, Veugelers P. Self-esteem and cognitive development in the era of the childhood obesity epidemic. Obes Rev. 2008; 9: 615-23. 18647242 10.1111/j.1467-789X.2008.00507.x

[14] Puhl RM, Heuer CA. Obesity stigma: important considerations for public health. Am J Public Health. 2010; 100: 1019-28. 20075322 10.2105/AJPH.2009.159491 PMC2866597

[15] Vander Wal JS, Mitchell ER. Psychological complications of pediat-ric obesity. Pediatr Clin North Am. 2011;58: 1393-401. 22093858 10.1016/j.pcl.2011.09.008

[16] Varni JW, Burwinkle TM, Seid M. The PedsQLTM 4.0 as a school population health measure: Feasibility, reliability, and validity. Qual Life Res. 2006; 15: 203-15. 16468077 10.1007/s11136-005-1388-z

[17] Schwimmer JB, Burwinkle TM, Varni JW. Health-related quality of life of severely obese children and adolescents. Jama. 2003; 289: 1813-9. 12684360 10.1001/jama.289.14.1813

[18] Pinhas-Hamiel O, Singer S, Pilpel N, Fradkin A, Modan D, Reich-man B. Health-related quality of life among children and adolescents: associations with obesity. Int J Obes (Lond). 2006; 30: 267-72. 16231035 10.1038/sj.ijo.0803107

[19] Riazi A, Shakoor S, Dundas I, Eiser C, McKenzie SA. Health-related quality of life in a clinical sample of obese children and adolescents. Health Qual Life Outcomes. 2010; 8: 1. 21078146 10.1186/1477-7525-8-134 PMC2998471

[20] Khodaverdi F, Alhani F, Kazemnejad A, Khodaverdi Z. The relationship between obesity and quality of life in school children. Iran J Public Health. 2011; 40: 96. 23113078 PMC3481768

[21] Kuczmarski RJ, Ogden CL, Guo SS, Grummer-Strawn LM, Flegal KM, Mei Z, et al. 2000 CDC Growth Charts for the United States: methods and development. Vital and health statistics Series 11, Data from the national health survey. 2002; 246: 1-190. 12043359

[22] Varny J, Varny BT, Seid M. The PedsQl-4 as a school population health measure:. feasibility, realibity, and validity. Qual Life Res. 2006; 15: 203-15. 16468077 10.1007/s11136-005-1388-z

[23] Rajmil L, Herdman M, de Sanmamed M-JF, Detmar S, Bruil J, Ravens-Sieberer U, et al. Generic health-related quality of life instruments in children and adolescents: a qualitative analysis of content. J Adolesc Health. 2004; 34: 37-45. 14706404 10.1016/s1054-139x(03)00249-0

[24] Varni JW, Seid M, Kurtin PS. PedsQL^™^ 4.0: Reliability and validity of the Pediatric Quality of Life Inventory^™^ Version 4.0 Generic Core Scales in healthy and patient populations. Med Care. 2001; 39: 800-12. 11468499 10.1097/00005650-200108000-00006

[25] Amiri P, Eslamian G, Mirmiran P, Shiva N, Jafarabadi MA, Azizi F. Validity and reliability of the Iranian version of the Pediatric Quality of Life Inventory^™^ 4.0 (PedsQL^™^) Generic Core Scales in children. Health Qual Life Outcomes. 2012; 10: 3. 22221765 10.1186/1477-7525-10-3 PMC3311062

[26] Hovsepian S, Qorbani M, Motlagh ME, Madady A, Mansourian M, Gorabi AM, et al. Association of obesity and health related quality of life in Iranian children and adolescents: the Weight Disorders Survey of the CASPIAN-IV study. Journal of pediatric endocrinology & metabolism: JPEM. 2017; 30: 923-9. 28809752 10.1515/jpem-2016-0402

[27] Pakpour AH, Yekaninejad MS, Zarei F, Hashemi F, Steele MM, Varni JW. The PedsQL Oral Health Scale in Iranian children: reliability and validity. Int J Paediatr Dent. 2011; 21: 342-52. 21489002 10.1111/j.1365-263X.2011.01130.x

[28] Chen X, Origasa H, Ichida F, Kamibeppu K, Varni JW. Reliability and validity of the Pediatric Quality of Life Inventory^™^ (PedsQL^™^) Short Form 15 generic core scales in Japan. Qual Life Res. 2007; 16: 1239-49. 17616837 10.1007/s11136-007-9230-4

[29] Gkoltsiou K, Dimitrakaki C, Tzavara C, Papaevangelou V, Varni JW, Tountas Y. Measuring health-related quality of life in Greek children: psychometric properties of the Greek version of the Pediatric Quality of Life InventoryTM 4.0 Generic Core Scales. Qual Life Res. 2008; 17: 299-305. 18080786 10.1007/s11136-007-9294-1

[30] Cremeens J, Eiser C, Blades M. Factors influencing agreement between child self-report and parent proxy-reports on the Pediatric Quality of Life Inventory^™^ 4.0 (PedsQL^™^) Generic Core Scales. Health Qual Life Outcomes. 2006; 4: 58. 16942613 10.1186/1477-7525-4-58 PMC1564004

[31] Jalali-Farahani S, Chin YS, Amiri P, Mohd Taib MN. Body mass index (BMI)-for-age and health-related quality of life (HRQOL) among high school students in Tehran. Child Care Health Dev. 2014; 40: 731-9. 23952615 10.1111/cch.12103

[32] Michel G, Bisegger C, Fuhr DC, Abel T. Age and gender differences in health-related quality of life of children and adolescents in Europe: a multilevel analysis. Qual Life Res. 2009; 18: 1147. 19774493 10.1007/s11136-009-9538-3

[33] Wynne C, Comiskey C, Hollywood E, Quirke MB, O'Sullivan K, McGilloway S. The relationship between body mass index and health-related quality of life in urban disadvantaged children. Qual Life Res. 2014; 23: 1895-905. 24473990 10.1007/s11136-014-0634-7

[34] Ul-Haq Z, Mackay DF, Fenwick E, Pell JP. Meta-analysis of the association between body mass index and health-related quality of life among children and adolescents, assessed using the pediatric quality of life inventory index. J Pediatr. 2013; 162: 280-6 e1. 22959137 10.1016/j.jpeds.2012.07.049

[35] Mitchell TB, Steele RG. The Effect of Body Mass Index, Negative Affect, and Disordered Eating on Health-Related Quality of Life in Preadolescent Youth. J Pediatr Psychol. 2016; 41: 768-76. 26791390 10.1093/jpepsy/jsv163

[36] Williams J, Wake M, Hesketh K, Maher E, Waters E. Health-related quality of life of overweight and obese children. Jama. 2005; 293: 70-6. 15632338 10.1001/jama.293.1.70

[37] Friedlander SL, Larkin EK, Rosen CL, Palermo TM, Redline S. Decreased quality of life associated with obesity in school-aged children. Arch Pediatr Adolesc Med. 2003; 157: 1206-11. 14662577 10.1001/archpedi.157.12.1206

[38] Fiveash L. The relationship among obesity, QOL, and health care in African American school children: PhD thesis]. The University of Alabama at Birmingham; 2003.

[39] Hughes A, Farewell K, Harris D, Reilly J. Quality of life in a clinical sample. 2007. 10.1038/sj.ijo.0803410 16733522

[40] Janssen I, Craig WM, Boyce WF, Pickett W. Associations between overweight and obesity with bullying behaviors in school-aged children. Pediatrics. 2004; 113: 1187-94. 15121928 10.1542/peds.113.5.1187

[41] Rapp-Paglicci LA, Dulmus CN, Wodarski JS. Handbook of preventive interventions for children and adolescents: Wiley; 2004.

